# Association of anthelmintic treatment with malaria prevalence in Rural Sussundenga, Mozambique

**DOI:** 10.12688/wellcomeopenres.19548.1

**Published:** 2023-09-20

**Authors:** Joseph A. Akambase, João L. Ferrão, Albino Francisco, Valy Muhiro, Anísio Novela, Dominique E. Earland, Kelly M. Searle

**Affiliations:** 1 Epidemiology and Community Health, University of Minnesota Twin Cities, Minneapolis, Minnesota, USA; 2 Engineering, UniSCED Aberta de Mozambique, Beira, Mozambique; 3 Escola Secondária de Sussundenga, Sussundenga, Mozambique; 4 Sussundenge-Sede Centro de Saude Rural, Sussundenga, Mozambique

**Keywords:** Malaria, epidemiology, interventions, anthelmintics

## Abstract

**Background:** Mozambique has the 4 ^th^ highest malaria incidence and mortality globally. Despite the existing malaria control strategies, malaria prevalence remains stagnant. These challenges have increased calls for innovative strategies in areas with the highest disease burden. Community mass treatment with anthelmintic agents have been used as an effective tool for the control of major helminth infections and has emerged as a potential tool for vector control in the fight against malaria.

**Methods:** This was an analysis of data from a cross-sectional community-based survey designed to study malaria risk, prevention, and health seeking behaviors in Sussundenga, Mozambique. Using logistic regression models, we quantified the association between ever receiving anthelmintic treatment and *P. falciparum* infection. We also fit models to determine the association between recent anthelmintic treatment and malaria infection.

**Results:** Two-hundred, seventy-seven (277) participants from 83 households were included in this analysis. The prevalence of *P. falciparum* infection measured by rapid diagnostic test (RDT) was 30%. 77% of participants reported having ever received anthelmintics. The prevalence of malaria was slightly higher among participants who reported ever taking anthelmintics. There was no statistically significant association between prior receipt of anthelmintic and *P. falciparum* malaria infection after adjusting for age, ITN use and head of household full-time employment (OR = 1.37, 95% CI, 0.70–2.70, p = 0.36). However, recent intake of anthelmintics was associated with lower odds of testing positive for in the adjusted models (OR = 0.35, 95% CI, 0.07–1.80, p = 0.21), but this was not statistically significant.

**Conclusions:** Our findings show that the benefit of anthelmintics treatment as a control tool for *P. falciparum* malaria infection is likely tied to when it is administered rather than if it was ever administered. These findings offer evidence for making decisions in planning mass community deworming in sub-Saharan Africa.

## Introduction

Malaria continues to be a serious global public health problem particularly in sub-Saharan Africa. Despite recent advances made in malaria control and elimination, an estimated 241 million cases of malaria were recorded in 2020 worldwide. The World Health Organization (WHO) African Region accounts for nearly 95% of global malaria cases ^1^. Mozambique alongside five other African countries including Nigeria, Burkina Faso, Uganda, Angola and the Democratic Republic of Congo account for over fifty percent (50%) of the total global malaria cases ^1^.

Several control and elimination strategies have been implemented with the goal of reaching the WHO’s Global Technical for Malaria (GTS) target of a 90% reduction in malaria incidence and mortality rates by 2030 ^2^. Control efforts have primarily focused on prevention, effective diagnosis and treatment, and expanding access to healthcare. Strategies used have included but are not limited to: use of insecticide treated bed nets (ITN), indoor residual spraying (IRS), early diagnosis and management of cases using artemisinin-based combination therapy (ACT) alongside integrated community care management (iCCM) and intermittent preventive treatment in pregnancy(iPTP) ^1, 3– 6^.

In spite of the proven efficacy of these existing malaria control strategies, the global prevalence of malaria continues to increase ^1^. This is likely due to challenges with implementation because of inadequate funding of malaria control programs in low- and middle-income countries in sub-Saharan Africa. It is also due to increasing vector resistance to insecticides ^7, 8^. These challenges have increased calls for innovative strategies particularly in areas with highest disease burden and to develop new and easy to implement control methods ^9^.

Community mass treatment with anthelmintic agents (i.e., dewormers) are community-based chemotherapy programs that have long been deployed as an effective tool for the control of major helminth infections in high burden communities ^10, 11^. The practice has emerged over the past few decades as a promising vector control tool for malaria following reports of *Anopheles* mosquitoes’ susceptibility to ivermectin in controlled trials ^12, 13^. Ivermectin is a specific anthelmintic used to control several parasitic infections in various settings across sub-Saharan African countries ^14^. Blood meals containing ivermectin have been reported to increase the mortality of *Anopheles* mosquitoes, reduce sporogony, and also delay refeeding frequency ^15– 17^. Similarly, prolonged periods of mass treatment with ivermectin have been found to be linked to reduced mosquito feeding ^18^. Many studies have largely reported on the effects of ivermectin on *Anopheles* mosquitoes with a few reporting on the effects of other groups of anthelmintics or community treatment with any anthelmintics. In addition, many of the reports of the effects of other anthelmintics groups including the benzimidazoles and Praziquantel on malaria prevalence in observational settings have largely been conflicting ^4, 19, 20^.

In this analysis, we explored the relationship between prior treatment with any anthelmintics and malaria risk in an observational study in the village of Sussundenga, Mozambique. We addressed this through three objectives: 1) identify the factors independently associated with *P. falciparum* malaria infection measured by rapid diagnostic test (RDT); 2) determine whether prior intake of any anthelmintic agent was associated with risk of malaria infection; and 3) determine whether the timing of anthelmintic treatment impacted the association between treatment and malaria risk.

## Methods

### Study design and population

This study was conducted using data obtained from a cross-sectional community-based survey designed to study malaria risk, prevention, and health seeking behaviors in Sussundenga, Mozambique from December 2019 – February 2020. Sussundenga village is the district capital of the Sussundenga District in the Manica Province, western Mozambique ^21^.

### Study area

The original study was conducted in the Sussundenga village. Sussundenga is a rural village, located in Sussundenga District, Manica Province, Mozambique. The village is approximately 70km from the Provincial capital of Chimoio, and 40km from the Zimbabwean border. The climate is tropical with an average annual precipitation of 1200 mm. There is a distinct rainy season that lasts from November – April, with a dry season from May – October ^19, 20^. The village is divided administratively in 17 residential areas called “Bairros” ^19^. This area has perennial malaria transmission, with seasonal increases in *P. falciparum* malaria incidence during and following the rainy season. Sussundenga village is the district capital where the central municipal offices and district level rural health center (RHC) reside. There is a central village for local commerce with primary and secondary schools. The local population is primarily agrarian with a population of approximately 20,000 inhabitants ^19, 20^.

### Data collection

**Figure 1.**
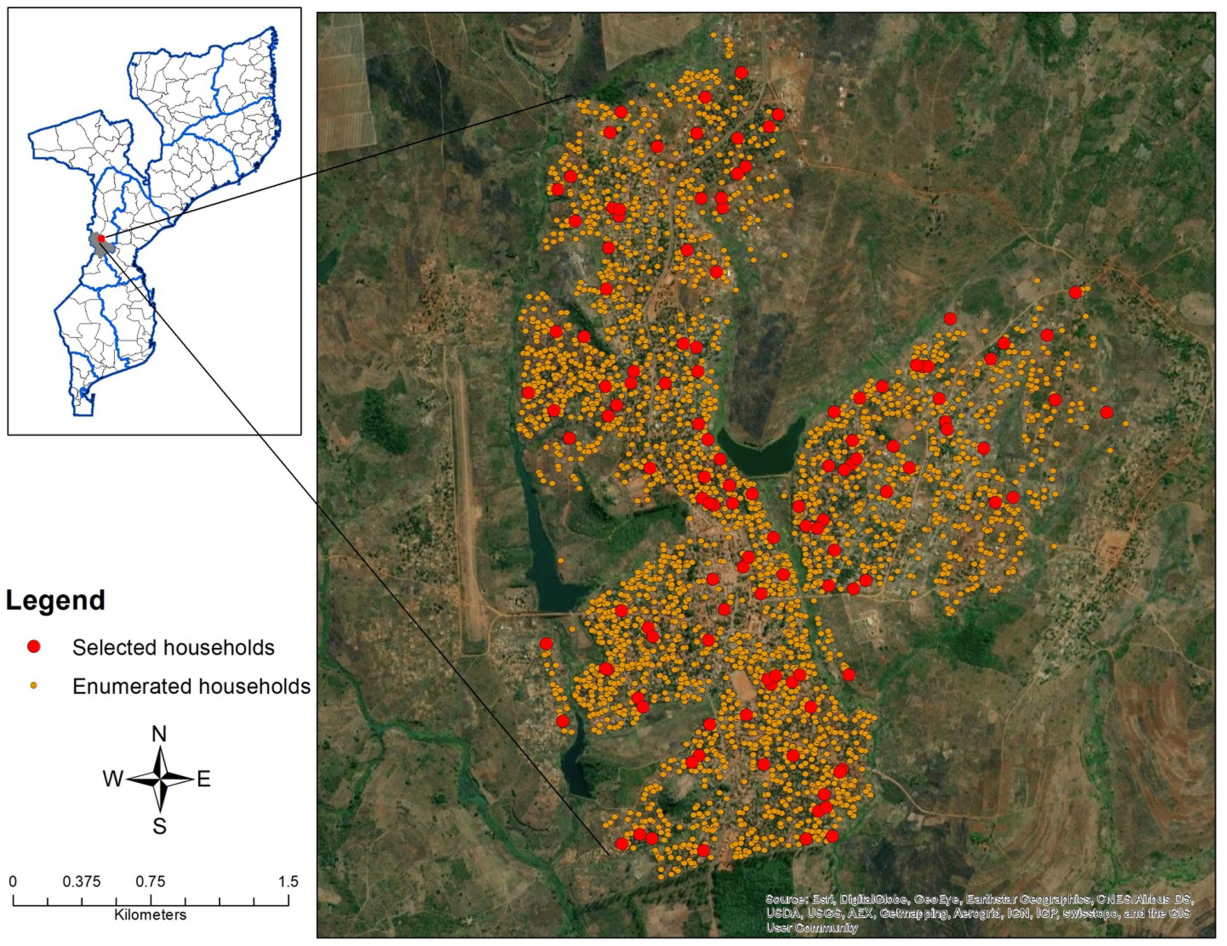
Map of the study area with enumerated and selected households.

GoogleEarth Pro ^TM^ satellite imagery was used to digitize and enumerate all household structures in the village of Sussundenga. A total of 2,837 households were identified in Sussundenga Village from the satellite imagery. A random sample of 125 households was taken with the goal of having 100 households for enrollment in the study and 25 households as backup for refusals and errors in the digitizing process (misclassified non-household structures) ( Figure 1). The sample was taken randomly to reduce selection bias. The sample size of 100 was calculated based on the assumption of 5-6 residents per household and to distinguish between individual risk factors for *P. falciparum* malaria infection among residents.

Coordinates of the households were extracted using a GoogleEarth Pro ^TM^ and maps of the selected households were created for the study team to conduct study visits. The study involved two visits to the selected households. The first was a notification visit where the study team introduced themselves to the head of the household and explained the objectives and procedures of the study. It is customary for the head of household to provide permission to the study team before any activities take place at the household involving other household members. Once the head of household gave permission, the study team conducted a household census with the head of household and began the process of individual informed consent with the household residents, for all adult (18+ years) residents and parental permission and assent from minors. After obtaining consent from the household residents, the study team informed participants when they would return the following day to conduct the study activities. The only eligibility requirement was that the residents live in household full time.

Data collectors administered a questionnaire to collect individual demographics (e.g. age, sex, education, and occupation), recent malaria symptoms, use of an ITN the previous night, and healthcare access and use. The questionnaire also included questions regarding administration and timing of anthelmintic treatment. The questionnaire was based on the Malaria Indicator Survey (MIS) with modifications for this study population. The questionnaire was piloted among the study team to assess understanding and validity of the questions prior to the start of the study. A study nurse collected current malaria specific symptoms by self-report. They then collected a finger prick blood sample to administer an (RDT) [RightSign Biotest ^®^, China]. The results were recorded and, in the event, that a participant was positive for malaria, the study nurse referred them to the Sussundenga RHC for diagnosis confirmation and treatment.

The questionnaire was conducted using tablet computers with the REDCap [Vanderbilt University, Nashville, USA] mobile application. Data was stored in a secure REDCap server hosted by the University of Minnesota.

### Ethical considerations

Ethical review and approval for this study was completed by the Institutional Review Board (IRB) at the University of Minnesota [STUDY00007184] on November 15, 2019 and from A Comissão Nacional de Bioética em Saúde (CNBS) at the Ministry of Health of Mozambique [IRB00002657] on November 11, 2019.

### Statistical analysis

Data was exported from REDCap where the original dataset was collected and stored. Descriptive statistics were used to report continuous variables by medians and interquartile range. Categorical variables were reported as proportions. Univariable logistic regression models were built to determine the associations between malaria infection by RDT and various participant characteristics. These models explored the independent associations between age, sex, ITN use, head of household occupation location (indoors vs. outdoors), head of household employment status (full-time vs. part-time), head of household education level, and sleeping outside of the household in the past month and malaria infection. Similarly, the association between those reporting any anthelmintic treatment and those reporting no anthelmintic treatment and malaria infection was investigated using a univariable logistic regression model. A multivariable model was also used to investigate the association between any anthelmintic use and malaria infection while controlling for confounders determined in the previous univariable models. In the multivariable model age, head of household full-time employment, and use of ITN were included to adjust for potential confounding. To investigate the association between timing of anthelmintic use and malaria infection additional univariable and multivariable models were constructed with the main exposure defined as taking anthelmintic treatment in the previous 6 months. This multivariable model was adjusted for age, ITNs use, and head of household full-time employment as confounders. Odds ratios with corresponding 95% confidence intervals were used to determine associations.

## Results

### Characteristics of participants

In total, 277 out of 309 participants from 83 households who responded to the survey were included in this analysis ^26^. Of the 277 respondents, 77% (214) reported having ever received anthelmintics. The median ages of participants who reported prior intake of anthelmintics and those who had not previously received anthelmintics were 18 (IQR 11 – 27) and 15 (IQR 3 – 25) years respectively. Among those who lived in a household where the head of household had full-time employment, 65% of participants had previously received anthelmintics compared to 60% of participants who had never received anthelmintics. Use of ITNs on the previous night was less prevalent among participants who previously received anthelmintics (62%) compared to participants with no history of anthelmintics intake (71%) ( Table 1).

**Table 1.** Basic characteristics of participants [% (n), Unless otherwise stated].

Characteristics	De-wormer (n = 214)	No De-wormer (n = 63)
Age [median (IQR)]	18 (11 – 27)	15 (3 - 25)
Sex (male)	45% (96)	48% (30)
Insecticide treated net use	62% (133)	71% (45)
Malaria prevalence by RDT	31% (66)	27% (17)
Head of household occupation (Indoor)	50% (107)	41% (26)
Head of household employment status (Full-time)	65% (140)	60% (38)
Head of household education level		
No education	11% (23)	13% (8)
Primary	29% (63)	40% (25)
Secondary	27% (57)	17% (11)
Tertiary	33% (71)	30% (19)
Slept outside of usual homes in the past month	29% (61)	35% (22)

### Prevalence of malaria

**Figure 2.**
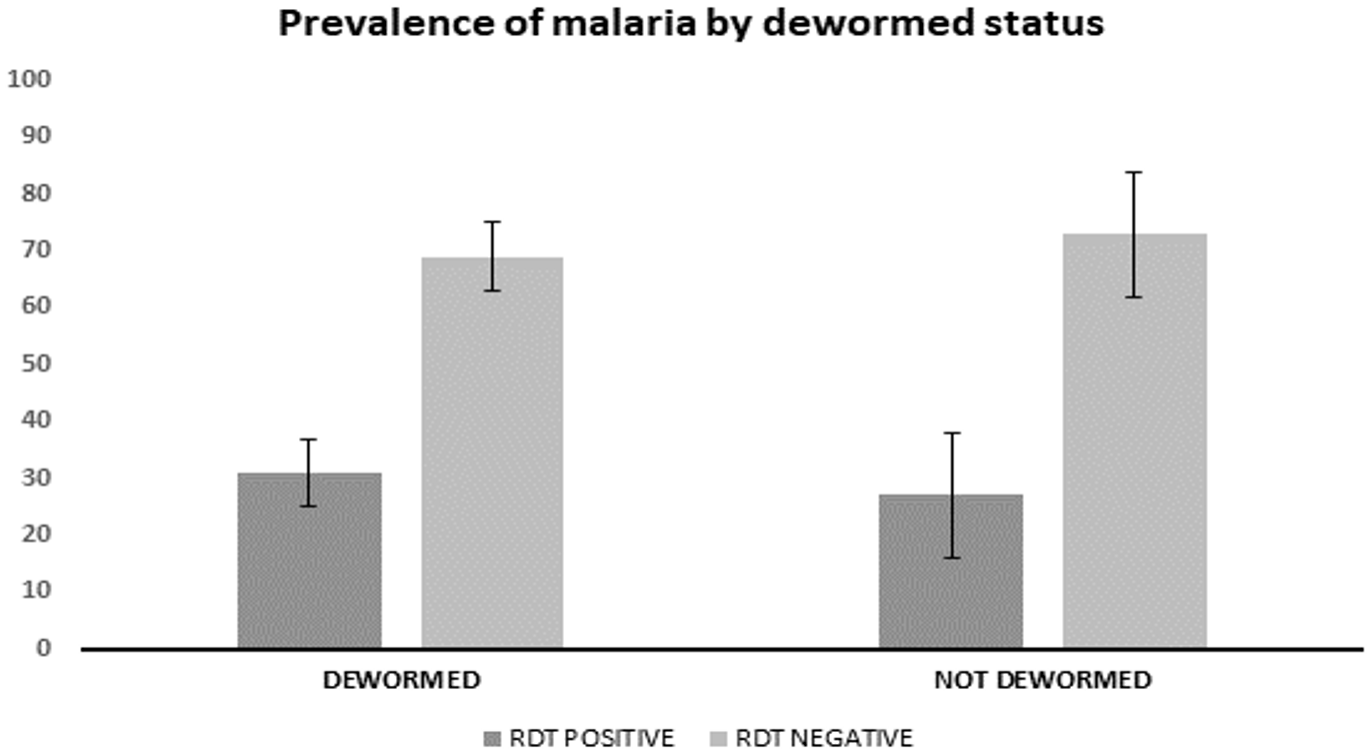
Malaria prevalence by deworming status.

The overall prevalence of *P. falciparum* infection was 30%. The prevalence of malaria was slightly higher among participants who reported previous intake of anthelmintics, 31% (66) compared to those who had never received anthelmintics, 27% (17) ( Figure 2).

### Predictors of *P. falciparum* malaria infection

The associations between various predictors and *P. falciparum* infection are presented in Table 2. Age, ITN use, and head of household’s full-time employment were independent predictors of *P. falciparum* infection. A one-year increase in age was associated with 3% lower odds of malaria infection (OR = 0.97, 95% CI, 0.95 – 0.99). Use of ITNs, and head of household’s full-time employment were respectively associated with 46% and 59% lower odds of malaria infection (OR = 0.54, 95% CI, 0.32 – 0.92; OR = 0.41, 95% CI, 0.24 – 0.69) ( Table 2).

**Table 2.** Predictors of *P. falciparum* malaria infection.

Predictors	OR (95 % CI)	p-value
Age (years)	0.97 (0.95 – 0.99)	**0.003**
Male (vs female)	1.09 (0.65 – 1.83)	0.742
Insecticide treated net use (vs non-use)	0.54 (0.32 – 0.92)	**0.023**
Head of household occupation (Indoor vs outdoor)	0.77 (0.46 – 1.29)	0.312
Head of household employment (Full-time vs part-time)	0.41 (0.24 – 0.69)	**0.001**
**Head of household education level** Primary (vs no education) Secondary (vs no education) Tertiary (vs no education)		
	1.09 (0.47 – 2.56)	0.842
	0.66 (0.26 – 1.63)	0.362
	0.55 (0.23 – 1.34)	0.189
Sleeping outside of their usual homes in the past month	0.93 (0.53 – 1.64)	0.803

### Association between deworming and the prevalence of malaria by RDT

The odds of *P. falciparum* infection was 1.21 times higher for those who had ever taken anthelmintics compared to those who have never taken anthelmintics in the unadjusted model, however this lacked statistical significance (95% CI, 0.64 – 2.26). In the model adjusted for age, ITN use and head of household full-time employment the odds of *P. falciparum* was 1.37 times higher among those who had ever taken anthelmintics compared to those who had not. The adjusted model also lacked statistical significance (95% CI, 0.70 – 2.70). ( Table 3).

**Table 3.** Relationship between deworming and *P. falciparum* malaria infection.

Exposure	OR (95% CI)	p-value	AOR (95% CI)	p-value
Dewormed (vs not dewormed)	1.21 (0.64 – 2.26)	0.56	1.37 (0.70 – 2.70)	0.36

*AOR – Adjusted odds ratio (Age, ITNs use and head of household full-time employment.

### Timing of dewormer administration and *P. falciparum* malaria infection

In the unadjusted model, recent (< 6 months) intake of anthelmintics was associated with a 0.44 times lower odds of *P. falciparum* infection compared to those with non-recent intake of anthelmintics. This result lacked in precision (95% CI, 0.09 – 2.09). In the model adjusting for age, ITNs use, and head of household full-time employment, recent intake of anthelmintics was associated with a 0.35 times lower odds of *P. falciparum* infection. This result also lacked precision (95% CI, 0.07 – 1.80) ( Table 4).

**Table 4.** Effect of timing of dewormer administration and *P. falciparum* malaria infection.

Exposure	OR (95% CI)	p-value	AOR (95% CI)	p-value
Dewormed within last 6 months (vs > 6months)	0.44 (0.09 – 2.09)	0.30	0.35 (0.07 – 1.80)	0.21

## Discussion

The overall prevalence of *P. falciparum* infection was 30%. Age of participants, use of ITNs, and head of household’s full-time employment were independent predictors of *P. falciparum* infection in Sussundenga village. We found that any prior receipt of anthelmintic was associated with a 21% increase in the odds of *P. falciparum* malaria infection. However, the association lacked in precision and had wide confidence intervals. Interestingly, this finding was inconsistent when the timing of anthelmintic administration was considered. Though our results lacked precision they showed that participants who received anthelmintics within the last 6 months had lower odds of *P. falciparum* infection compared to those that received anthelmintics over 6 months prior to the survey.

Similar to our findings, Dila *et al*. in a meta-analysis found no significant association between treatment with anthelmintics and malaria prevalence at the end of follow up period (pooled OR 0.93, 95% CI: 0.62 - 1.38) ^19^. Contrary to these findings, Sokhna *et al*. found an increase in malaria incidence among children presenting with concomitant helminths infection ^22^. Thereby, implying that an anthelmintic treatment was potentially associated with malaria risk in these settings. In 2014, Salazar-Castañon *et al*. determined that chronic helminthic infections increase an individual’s susceptibility to acute malaria by causing a major shift in the host immune response from Th1 to Th2 ^23^. Furthermore, Salazar-Castañon *et al*. maintained that treatment with anthelmintics may also trigger a shift of the immune response to Th1 which ultimately lead to a decreased host’s susceptibility to acute malaria infection ^23^. The timing of anthelmintics administration as well as the duration of helminth infection have emerged as critical determinants of a host’s immune response to *P. falciparum* parasites. Consistent with the effects of timing of anthelmintics administration, we found that participants who received anthelmintics within a six-month period had lower odds of *P. falciparum* infection compared to those that received anthelmintics more than six-months prior, though this finding was not statistically significant. We suspect the protective benefit of anthelmintics treatment may have waned considerably over time. Furthermore, the imprecision of our estimates were likely to have been from our small sample size as well as differences in mechanism of action and effects of the various anthelmintics that were received by participants on the overall immune response. Additionally, our study did not collect information on the different types of anthelmintics that were received by participants and the frequency of treatment. Previous studies had found that repeated 2 – 4-monthly anthelmintic treatments can have a significant impact on *Plasmodium* infection ^24, 25^.

Our study has several limitations. First, this was a community-based cross-sectional survey and by design we were limited in our ability to establish any true causal relationship. Second, our small sample size of 277 may not have been large enough to detect true differences in this association as it was not powered for this analysis. Third, we collected no information on specific classes of anthelmintics and dosages of anthelmintics received by the participants. This is important as pharmacological effects as well as effects on the level of immune responses could differ depending on the type of anthelmintics received. Lastly, malaria infection was measured by RDT only without confirmation with microscopy or polymerase chain reaction (PCR). This is a high transmission setting and some of the infections detected by RDT in this study were asymptomatic, but most were moderately symptomatic. However, it is likely that low density infections were missed using only the RDT.

## Conclusions

Age, use of ITNs, and heads of households’ full-time employment status were associated with *P. falciparum* infection in Sussundenga. We also showed that the potential benefit of anthelmintics treatment as a control tool for *P. falciparum* malaria infection is most likely tied to when it is administered rather than if they were ever administered, though our findings were imprecise. These findings offer evidence for making decisions in planning mass community deworming and to inform the overall objectives of such policies.

## Data Availability

Dryad: Association of Anthelmintic Treatment with Malaria Prevalence in Rural Sussundenga, Mozambique. https://doi.org/10.5061/dryad.79cnp5hx1 ^26^. Data are available under the terms of the Creative Commons Zero "No rights reserved" data waiver (CC0 1.0 Public domain dedication).
