# Supplementary material for: Association of anthelmintic treatment with malaria prevalence in Rural Sussundenga, Mozambique
Source: Wellcome Open Res. 2023 Sep 20;8:417. [Version 1] doi: 10.12688/wellcomeopenres.19548.1 (PMC11018338; doi:10.12688/wellcomeopenres.19548.1)
Supplement: Peer review history

# Reviewer report: Reviewer response for version 1

**DOI:** 10.21956/wellcomeopenres.21654.r71262

**Published:** 2024-04-09

**Authors:** Poe Poe Aung

**Affiliations:** 1 CAPHRI, Maastricht University Medical Center, Maastricht University, Maastricht, The Netherlands; 2 Malaria Consortium, Bangkok, Thailand

**Reviewer comment**

Summary of the research and overall impression

 This manuscript aimed to identify the prevalence of malaria and factors associated with malaria infection, in relation to the use of deworming in Mozambique. The paper is well presented and a few minor comments are mentioned below.

Discussion on specific area of improvement

 Abstract

1. Abstract is presenting clearly in study design and methodology, data analysis methods, key findings of prevalence and factors associated with high malaria burden in Mozambique. No further comment for the abstract.

Introduction

1. 2 ^nd^ paragraph: iCCM is meant for “integrated community case management” I think. Pls double check you are mentioning “integrated community care management”.  
2. World Malaria Report data should be updated with the latest version of the report, which is 2023.

Methods - none

 Results

1. P-value should be added in table 1 and figure 2.

Discussion - none

 Conclusion - none

Is the work clearly and accurately presented and does it cite the current literature?

Yes

If applicable, is the statistical analysis and its interpretation appropriate?

Yes

Are all the source data underlying the results available to ensure full reproducibility?

Yes

Is the study design appropriate and is the work technically sound?

Yes

Are the conclusions drawn adequately supported by the results?

Yes

Are sufficient details of methods and analysis provided to allow replication by others?

Yes

Reviewer Expertise:

public health, malaria, sexual and reproductive health, maternal child health, lymphatic filariasis, WASH

I confirm that I have read this submission and believe that I have an appropriate level of expertise to confirm that it is of an acceptable scientific standard.

# Reviewer report: Reviewer response for version 1

**DOI:** 10.21956/wellcomeopenres.21654.r69481

**Published:** 2024-04-09

**Authors:** Nkemngo Francis Nongley, PhD

**Affiliations:** 1 Infection and Disease Control Unit, Centre for Infection Biology and Tropical Health (CIBITH), Forzi Institute, Buea, Southwest, Cameroon

**General synopsis:**

 The study by Akambase attempted to report on the association between anthelminthic treatment and malaria prevalence in a locality in Mozambique. Limited by the small sample size, the study assessed the prevalence of malaria among dewormers (6 months after anthelminthic intake) and non-dewormers using a less sensitive detection method (RDT), revealing similar frequency levels of P. falciparum infection. Moreover, the authors employed a model to assess the link between recent and over-time intake of anthelminthic treatment and malaria infection, observing a lower odds of acquiring P. falciparum infection in the recent treatment groups. However, this decrease was NOT statistically significant between the two groups.

 Conflicting results exist in the literature on this topic, particularly about helminths increasing or decreasing the risk of malaria infection. Beaming on the effect of anthelminthic treatment on malaria prevalence is an interesting subject matter, particularly to partly address the conflict on helminths and malaria outcomes. The study tried to answer the question of whether the timing of anthelminthic treatment influences a skew in malaria prevalence, although no strong causal relationship was established. Importantly, the authors outlined the limitations of the study, including study design, sample size, diagnostic test sensitivity, and no data on the type of anthelminthic drug administered to the community. I have made major and significant comments on the MS for the authors to address. Please see below:

**Conclusion of the abstract:** This technically sounds obvious because the concentration of the anthelminthics will decay over time (and be eliminated from the body) to sub-therapeutic levels, thus exhibiting a minimal effect on new or ongoing Pf infections.

**Introduction:**

 First paragraph: Please update the morbidity and mortality data from the recently published world malaria report (WHO, malaria report 2023)

 Third paragraph: Modify the statement to “increasing vector and parasite resistance to insecticides and antimalarial drugs, respectively.”. Also, the last statement is incomplete. Correct it to develop new and easy "strategies" to “complement” control methods

**Method section:**

 Data collection: Please could you comment if 100 is the total number of individuals sampled or the number of households included in the study. I find it difficult to understand because if 100 is the overall number of 5-6 residents per household, then the sample size is small and weakens the overall study design and data generated.

 What was the antimalarial treatment history of the 83 household members? Noting that Sussundenga has a high community prevalence of malaria, it suggests that individuals will frequently be sick of malaria and, as such, will take treatment to alleviate symptoms. Therefore, it is important to unbiasely rule out the effect of previously antimalarial treatment intake as a confounder relative to the independent, measurable impact of antihelminthics on malaria burden in this locality.

 Data collectors administered a questionnaire. The questionnaire failed to include a history of antimalarial medication.

**Results section:**

 Prevalence of malaria: Specify the prevalence in relation to the diagnostic method used (in this case, RDT). Please, can the authors’ comment explain why the gold standard microscopy was not employed?

 Table 1: The numerical difference between dewormers is 3x larger than that between non-dewormers. This is a significant bias, as it is difficult to pair-wise compute the effect of deworming on malaria infection and parasitemia.

 Secondly, looking at the age indicator, why did the authors not mainly focus on school-age children (3–15 years) for both subject categories (dewormers and ND). Indeed, MDA-based preventive chemotherapy (Mebendazole for STH) often targets this age group, and where certainty exists on deworming history for a 6-month interval period (at least by DOTS),. Including individuals over 15 years old in your study is a weakness, as a verbal record of taking anthelminthics may not be a true indication of a deworming history. Also, the minimum age range of dewormers (11-27) is far higher than the minimum for non-dewormers (3-35), and this is a potential confounder.

 This weakness strongly reflects on the malaria prevalence by RDT, where dewormers document a similar prevalence than their counterpart, posing a weakness on the MS title and overall conclusion of the manuscript itself. Please, critically, provide a convincing response to address these remarks.

 Does this imply that anthelminthics treat/clear P. falciparum infection? RDT is a less sensitive method to diagnose malaria, particularly in low parasite loads (as may coincide with the recent intake of anthelminthic drugs). Moreover, what was the antimalaria treatment history?

 Figure 2: I am certain that recent deworming will exhibit additional effects both on reducing STH egg burden and on lessening malaria incidence and prevalence. However, the data reported in this study is largely weakened by the study design and randomization. Indeed, by focusing on individuals who had been dewormed for at least 6 months prior to the study, would the deworming have any effect on malaria incidence after 6 months of deworming? This is clearly seen in Fig. 2, as I may be of the opinion that the malaria positivity status (of dewormed participants) could be attributed to recent infection that would not have been prevented by the 6-month post-deworming effect.

 Timing of dewormer administration and *P. falciparum* malaria infection: How was the modelling done? Please provide enough details on this. This aspect of this study is of major interest as it constitutes the basis of your manuscript title and abstract. However, very limited information is provided. Furthermore, the authors indicated the unadjusted model considered in account a <6-month interval of anthelminthics treatment. Please could the authors provide additional analysis to show the varied impact of recent intake of anthelminthics on malaria infection (with highlights on the antihelminthic malaria reduction effects of 1 week, 2 weeks, 1 month, 2 months, 3 months, or 5 of treatment). Also, I will be interested to see a logistic regression plot that depicts the impact of these varied anthelminthic treatment periods on malaria prevalence levels. This will provide strong evidence to replicate the data and also consider the validity of the results

**Discussion section:**

 Second paragraph: Please clarify if the Sokhna et al. study reported on the status of anthelminthic treatment as well or if the study only focused on the association between both infections. This will strengthen the following statement, where you are implying that helminthic treatment enhances the risk of malaria infection. Clarify appropriately

 Last paragrapgh: Although the authors highlighted this as a limitation, it will nevertheless be worthwhile to know what type of antihelminthic drugs were administered for deworming programs in this community. Was dewoming done annually or biannually? Aside from progammatic deworming campaigns, did participants document just receiving anthelminthis randomly to treat worm infections? Please comment on this.

Is the work clearly and accurately presented and does it cite the current literature?

Yes

If applicable, is the statistical analysis and its interpretation appropriate?

Partly

Are all the source data underlying the results available to ensure full reproducibility?

Yes

Is the study design appropriate and is the work technically sound?

No

Are the conclusions drawn adequately supported by the results?

Partly

Are sufficient details of methods and analysis provided to allow replication by others?

Yes

Reviewer Expertise:

Molecular epidemiology, Pathogen Genetics, Infection biology and Disease Control

I confirm that I have read this submission and believe that I have an appropriate level of expertise to confirm that it is of an acceptable scientific standard, however I have significant reservations, as outlined above.

# Reviewer report: Reviewer response for version 1

**DOI:** 10.21956/wellcomeopenres.21654.r71268

**Published:** 2024-04-09

**Authors:** Olawunmi Oyerinde

**Affiliations:** 1 Department of Biological Sciences, Mountain Top University, Ibafo, Ogun State, Nigeria

The article reports on the factors associated with reduced odds of malaria infection among the study population namely age, the use of insecticide treated nets (ITNs) and full-time employment of the household head. The authors further attempted to evaluate the significant association between use of anthelminthic drugs and *P. falciparum *infection.

 Generally, the manuscript is  well-written with a detailed background of the study provided. The study area is clearly described and the objectives of the study well outlined.

 However, the results and discussion sections should be reviewed.

 1. Table 1 can be more detailed to include the significance in proportion of those who had taken de-worming tablets and those who had not by running chi-square test. 

 2. Figure 2 may not be required as the information provided is already in Table 1. 

 3. For a more robust data, the authors could obtain the malaria diagnosis result by microscopy from the Sussundenga RHC since RDT positive patients were referred to the clinic.

 4. The interpretation of the association between deworming and prevalence of malaria by RDT should be with caution since no significant association was found. Hence, the conclusion of the abstract and the discussion section should be reviewed.

Is the work clearly and accurately presented and does it cite the current literature?

Yes

If applicable, is the statistical analysis and its interpretation appropriate?

Partly

Are all the source data underlying the results available to ensure full reproducibility?

Yes

Is the study design appropriate and is the work technically sound?

Yes

Are the conclusions drawn adequately supported by the results?

No

Are sufficient details of methods and analysis provided to allow replication by others?

Yes

Reviewer Expertise:

Epidemiology and immunology of malaria and co-infections

I confirm that I have read this submission and believe that I have an appropriate level of expertise to confirm that it is of an acceptable scientific standard, however I have significant reservations, as outlined above.

# Reviewer report: Reviewer response for version 1

**DOI:** 10.21956/wellcomeopenres.21654.r78473

**Published:** 2024-04-09

**Authors:** Zachary R Popkin-Hall

**Affiliations:** 1 The University of North Carolina at Chapel Hill, Chapel Hill, North Carolina, USA

Akambase *et al.* present an interesting and relevant analysis exploring whether the widespread use of antihelminthic agents have any impact on malaria prevalence in the rural Mozambican village of Sussundenga. Drawing on data that were generated for a cross-sectional community survey, they seek to determine 1) what factors are associated with *Plasmodium falciparum* positivity by RDT, 2) if antihelminthics are associated with *Pf *positivity and 3) if the timing of antihelminthic treatment is associated with *Pf *positivity. These are important questions to address, particularly in light of the body of literature that suggests that mass drug administration of ivermectin may decrease malaria prevalence and I am glad to see them explored here.

 The analysis presented here is interesting and largely well-written, but incomplete and in need of significant revision.While I understand that it is more challenging to write a coherent narrative from non-significant findings, parts of the manuscript are written as though the authors did obtain significant results and should be revised to reflect the actual findings. In addition, greater context for this study would be useful and informative, as would a greater exploration of alternative hypotheses.

**Major Comments**

 1. The text of the manuscript should be reworked to reflect the results that were actually found by Akambase *et al.*, not the hypotheses. As noted by the other reviewers, the conclusion of the abstract as well as the conclusion section of the actual manuscript currently suggest that timing of antihelminth treatment is associated with malaria positivity, when this is not reflected by the data analyses.

 2. I would have liked to see a more detailed summary of the body of literature showing whether ivermectin and other antihelminthic agents impact malaria positivity and prevalence. The discussion notes that different studies have identified conflicting results, while the introduction largely reports positive results. I think it will serve the authors' study better to engage more thoroughly with what the existing evidence actually says. Doing so will also better situate their own findings within the current scientific consensus.

 3. The age of study participants is a significant predictor of *Pf *positivity, as shown in Table 2. My brief survey of the literature suggests that this is also true for helminthic infections. I appreciate that the authors added age as a variable in their second model, however I think its impact is under-explored in the manuscript.

 In addition to using age as a continuous explanatory variable for the likelihood of malaria infection, I would suggest also treating it as a categorical variable (i.e. under-fives, schoolchildren, adults or children vs. adults) to see if there are any more significant impacts on children specifically. The malaria literature indicates that schoolchildren tend to have higher malaria prevalence than adults or younger children. As such, it stands to reason that they would also be the most likely to benefit from a reduction in prevalence associated with being treated for helminth infections.

**Minor Comments**

 1. While it is true that implementation challenges and insecticide resistance contribute to ongoing and in some cases increased malaria transmission, there are other factors to consider as well. I would suggest mentioning parasite factors such as treatment failure or delayed parasite clearance due to artemisinin and/or partner drug resistance, the spread of diagnostic resistance due to *Pfhrp2/3 *deletion, and potential increases in non-falciparum malaria species in response to intensive *Pf *control.

 2. The last two sentences of the fourth paragraph of the introduction are unclear. A more thorough summary of the literature on ivermectin/antihelminthic agents and their relationship to malaria prevalence/mosquito biting would help clarify.

 3. There are some very minor grammar issues in the Methods section, including an extraneous "a" article and unnecessary parentheses around RDT.

 4. The authors do a good job of exploring the limitations of their study, and it is clear that this is an add-on exploratory analysis. However, I think this section could be improved. The small sample size and lack of data collection on antihelminthic treatment are major impediments to drawing broad conclusions from this data, so I would suggest a more cautious interpretation than is currently provided.

 As noted by the other reviewers, it would be useful to see the power calculations used in this study. Providing this information would also help the authors better characterize the likelihood of finding a significant effect in a larger data set.

 Relying entirely on RDTs will undoubtedly result in an underestimate of *Pf *prevalence (given that subpatent infections will not be detected) in addition to preventing the detection of single-species infections with other *Plasmodium *parasites. I assume PCR or microscopy data is not available, so would suggest additional emphasis on this limitation.

 5. Given that this analysis relies on a small sample set and does not detect a significant effect of antihelminth treatments on *Pf* prevalence, I think the manuscript would be strengthened by exploring further analyses that could be performed to provide a more conclusive answer to this question. I would also suggest further situating the present study within the larger body of work on this question - how does this study site compare to others? Is there reason to think e.g. ivermectin MDA would have a larger or smaller effect here vs. elsewhere?

Is the work clearly and accurately presented and does it cite the current literature?

Partly

If applicable, is the statistical analysis and its interpretation appropriate?

Partly

Are all the source data underlying the results available to ensure full reproducibility?

Yes

Is the study design appropriate and is the work technically sound?

Partly

Are the conclusions drawn adequately supported by the results?

Partly

Are sufficient details of methods and analysis provided to allow replication by others?

Yes

Reviewer Expertise:

Dr. Zachary R. Popkin-Hall is a malaria and mosquito biologist whose research focuses primarily on non-falciparum malaria biology, genomics and genomic epidemiology in sub-Saharan Africa, particularly Cameroon and Tanzania.

I confirm that I have read this submission and believe that I have an appropriate level of expertise to confirm that it is of an acceptable scientific standard, however I have significant reservations, as outlined above.

# Reviewer report: Reviewer response for version 1

**DOI:** 10.21956/wellcomeopenres.21654.r75724

**Published:** 2024-04-08

**Authors:** Manju Rahi, Srikanth S

**Affiliations:** 1 ICMR- Vector Control Research Centre, Puducherry, Puducherry, India

Review of "Association of Anthelmintic Treatment with Malaria Prevalence in Rural Sussundenga, Mozambique"

 Is the work clearly and accurately presented, and does it cite the current literature?

 The manuscript is partly clear and accurately presented. The authors have made a commendable effort to contextualize their study within the current literature. However, further clarity in presenting the study's findings in alignment with cited literature would enhance the manuscript's contribution to the field.

 Is the study design appropriate, and is the work technically sound?

 The study design, a cross-sectional survey, is partly appropriate for exploring the association between anthelmintic treatment and malaria prevalence. While inherent limitations exist in inferring causality from such a design, the approach is technically sound for initial investigations. Future studies could build on this work with longitudinal designs to explore causality.

 If applicable, is the statistical analysis and its interpretation appropriate?

 The statistical analysis is partly appropriate, with a commendable effort made to analyze and interpret complex data. A more detailed exploration and clearer presentation of the statistical methods and interpretations, especially regarding non-significant findings, would provide deeper insights.

 Are the conclusions drawn adequately supported by the results?

 The conclusions are cautiously drawn and partly supported by the results. The authors acknowledge the limitations of their findings, which is commendable. A more explicit discussion o how the results inform the conclusions, especially in light of the study's design and the non-significant associations found, would further solidify the manuscript's contributions.

 Summary:

 The study by Akambase et al. provides valuable insights into the complex relationship between anthelmintic treatment and malaria prevalence. Their dedication and thoroughness of the authors are evident throughout the study. While there are areas for further clarification and enhancement, the effort to investigate this association in a challenging public health context is commendable. This work lays a foundation for future research that can explore these associations more deeply, potentially informing public health strategies in malaria-endemic regions. The authors are to be commended for their contribution to this important area of public health research, and their work encourages continued exploration of innovative strategies for malaria control.

**Overall Assessment**

**Introduction and Appreciation:** The study by Akambase et al., conducted as a cross-sectional community-based survey in Rural Sussundenga, Mozambique, investigates the association between anthelmintic treatment and P. falciparum infection, addressing an important public health question. The authors' effort to explore innovative strategies for malaria control in a region burdened by this disease is commendable and contributes valuable insights to the field. We appreciate the dedication behind this research.

**Scientific Rigor and Validity:** The study was a cross-sectional community-based survey aiming to "quantify the association between ever receiving anthelmintic treatment and P. falciparum infection." Despite this clear objective, the article acknowledges that "there was no statistically significant association between prior receipt of anthelmintic and P. falciparum malaria infection" (adjusted OR = 1.37, 95% CI 0.70–2.70, p = 0.36). This discrepancy between the lack of significant findings and the strong concluding statements about the potential benefits of anthelmintic treatment highlights a potential gap. The design and methodology, while adequate for observational insights, are insufficient for the causal interpretations suggested in the conclusions.

- **"There was no statistically significant association between prior receipt of anthelmintic and P. falciparum malaria infection"**: This quote underscores the need for caution in interpreting the study's outcomes. The discussion and conclusions sections should have reflected the tentative nature of the findings more clearly.

**Relevance and Originality:** The topic is undoubtedly relevant and offers a novel angle on malaria control strategies. However, the originality of the study is overshadowed by its limitations and the overinterpretation of its results. The claim that "the benefit of anthelmintics treatment as a control tool for P. falciparum malaria infection is likely tied to when it is administered" is speculative given the non-significant findings. Also, the malarial parasite infection occurs on mosquito bites and the half-life of anti-helminthic medications is between a few hours to a few days. Therefore studying the timing of the anti-helminthic consumption in 6-month intervals does not add to the research question.

**Methodological Concerns:** The cross-sectional design limits the ability to infer causality from the observed associations. There is a lack of a clearly defined temporal sequence between anthelmintic exposure and malaria outcome measurement.

 Additionally, the selection of the sample size and the statistical methods for controlling confounding variables are not sufficiently justified, impacting the reliability of the findings. The use of RDT alone in a study with objectives to associate anti-helminthics with malaria is a significant drawback in methodology.

 A formal power calculation could have been provided to justify the sample size for detecting hypothesized effects. With only 277 participants, the study may likely be underpowered, especially for subgroup analyses. Wide confidence intervals around effect estimates indicate a lack of precision due to a limited sample. There is a potential for unmeasured/residual confounding given the observational nature of the study. Since only 83 of 125 selected households agreed to participate, selection bias is also a concern. Reliance solely on RDTs may have led to outcome misclassification. Sole reliance on self-reported anthelmintic use may be subject to recall bias and misclassification.

**Clarity and Quality of Presentation:** The article is generally well-structured, with clear sections delineating the introduction, methods, results, and discussion. However, there are inconsistencies in terminology and a need for more clarity in data presentation. These issues, combined with the overinterpretation of non-significant results in the discussion and conclusions, detract from the overall presentation. There is a lack of a clear advance over prior research or a compelling new perspective offered. There is also no clearly defined knowledge gap that this study aims to address.

**Generalizability and Application:** The study's findings are presented without adequate discussion on their generalizability beyond the specific setting of Rural Sussundenga, Mozambique. This is crucial for understanding the broader implications of the research and its applicability to malaria control strategies in other contexts. But this is a commendable effort in the currently described setting.

**Ethical Considerations:** The article notes ethical approval from appropriate boards, indicating adherence to standard research ethics. This aspect is adequately addressed. The adherence to ethical guidelines and the provision of data availability statements are commendable practices that should be continued in future work.

**References and Contextualization:** The study situates its research within the broader context of malaria control efforts and the potential role of anthelmintic treatments. Yet, it occasionally overstates the implications of its findings without sufficient support from the data or existing literature. The limitations have been listed twice (at the end of the discussion paragraph and in the limitations sections). The findings from Dila et al., do not substantiate this study and Sokhna et al., also did not provide any valid evidence or discussion points. The Salazar-Castañon et al., paper also does not bring validity to a 6-month check on anti-helminthic efficacy. The limitations have been listed succinctly portraying the drawbacks of the study. Conclusions are not sufficiently grounded in the limitations of the study design and findings.

-
  - **"Our findings show that the benefit of anthelmintics treatment as a control tool for P. falciparum malaria infection is likely tied to when it is administered"**: Given the non-significant results, this statement may mislead readers regarding the study's implications. Future manuscripts should ensure claims are substantiated by the data.

**Suggestions for Improvement:**

- **Methodological Enhancements:** Future research should aim for designs that can better establish causal relationships, accompanied by larger sample sizes and more rigorous statistical analysis.
- **Data Interpretation:** The conclusions drawn should more accurately reflect the limitations and uncertainties inherent in the study's findings. Avoid overstating the significance of non-significant results.
- **Literature Integration:** A more cautious and balanced discussion of the findings within the current research landscape would strengthen the paper. This includes acknowledging conflicting evidence and the speculative nature of certain conclusions.
- **Other corrections:** The title claiming an "association" is not accurate based on the non-significant findings. A more neutral title would be preferable. The abstract and conclusions may avoid overstatements about the findings and stick to simply reporting the lack of significant associations found. Use consistent tense throughout - future or present for describing study aims/design and past tense for reporting methods and results.

**Conclusion**

 While the study addresses an intriguing hypothesis about anthelmintic treatment and malaria prevalence, the leap from the observed non-significant associations to the implications for malaria control strategies seems premature.

 Future editions should aim to demonstrate statistical significance where applicable, thoroughly justify the chosen methodology, and discuss the findings' relevance and applicability to broader public health strategies. We believe that addressing these points will make a substantial contribution to the manuscript's strength and adjustments in the interpretation of results and a cautious approach to conclusions would enhance the manuscript's scientific contribution.

Is the work clearly and accurately presented and does it cite the current literature?

Partly

If applicable, is the statistical analysis and its interpretation appropriate?

Partly

Are all the source data underlying the results available to ensure full reproducibility?

Yes

Is the study design appropriate and is the work technically sound?

Partly

Are the conclusions drawn adequately supported by the results?

No

Are sufficient details of methods and analysis provided to allow replication by others?

Yes

Reviewer Expertise:

Dr. Manju Rahi, currently the Director and Scientist 'G' at the Vector Control Research Centre, Puducherry, has a rich expertise in malaria research, epidemiology, and the strategic implementation of antihelminthic treatments in public health programs. Her extensive experience is supported by an academic foundation in Preventive and Social Medicine and by her ongoing PhD research in Malaria Epidemiology. This supports her profound understanding of the dynamics and control strategies of vector-borne diseases. Dr. Rahi has played a pivotal role in coordinating national policy translation studies, including the integration of DEC salt in lymphatic filariasis control and pioneering Wolbachia-based vector control strategies. Having contributed significantly to research that shapes national policies and guidelines, Dr. Rahi's involvement in assessing the scientific merit of works within these domains is critical. Her roles as a reviewer for esteemed journals demonstrate her commitment to advancing public health through rigorous scientific inquiry and evidence-based interventions. Given this comprehensive background, Dr. Rahi is well-positioned to critically evaluate manuscripts, ensuring they meet the scientific standards required for advancing the understanding and control of vector-borne diseases.

We confirm that we have read this submission and believe that we have an appropriate level of expertise to state that we do not consider it to be of an acceptable scientific standard, for reasons outlined above.

# Reviewer report: Reviewer response for version 1

**DOI:** 10.21956/wellcomeopenres.21654.r75739

**Published:** 2024-04-08

**Authors:** Adilson DePINA

**Affiliations:** 1 CCS-SIDA, Ministério da Saúde e da Segurança Social, Praia, Cape Verde

**Association of anthelmintic treatment with malaria prevalence in Rural Sussundenga, Mozambique**

**REVIEW**

  The Introduction section contains data and references that do not reflect the latest information available, e.g. case of malaria worldwide refer to 2020.  The authors should cite data from the 2023 World Malaria Report (2022 estimates 249 million cases).

 The authors state in the Introduction section that that, “global prevalence of malaria continues to increase,” and cites the 2021 World Malaria Report.  The global prevalence is not increasing, so  this  statement needs to be corrected.

 The authors describe their sample size, but not provide a power calculation and underlying assumptions.  This is needed to be clarified.

*Plasmodium falciparum* should be written out in full the first time it is used and abbreviated as *P. falciparum* thereafter.  This should be reviewed in the all main body of the text. 

 In general, the manuscript is very interesting, the redaction is good but need some improvement to better comprehension, before the acceptation to publication. Some suggestion of correction:

***Abstract***

 Community mass treatment with anthelmintic agents have been used as an effective tool for the control of major helminth infections it has emerged  It has emerged…

 This was an analysis of data from a cross-sectional community-based survey designed to study malaria risk, prevention, and health seeking behaviors in Sussundenga, Mozambique. 

 Using logistic regression models, we quantified the association between ever receiving anthelmintic treatment and P. falciparum infection. We quantified the association between ever receiving anthelmintic treatment and P. falciparum infection using logistic regression models.

 With lower odds of testing positive for in the adjusted models (OR = 0.35, 95% CI, 0.07–1.80, p = 0.21) 

***Introduction***

 Malaria continues to be a serious global public health problem particularly in sub-Saharan Africa

 Mozambique alongside five other African countries including Nigeria, Burkina Faso, Uganda, Angola and the Democratic Republic of Congo account for over fifty percent (50%) of the total global malaria cases. Mozambique, along with five other African countries, including Nigeria, Burkina Faso, Uganda, Angola, and the Democratic Republic of Congo, accounts for over fifty per cent (50%) of the total global malaria cases.

 Strategies used have included but are not limited to: use of insecticide treated

 bed nets (ITN),

 In spite of the proven efficacy of these existing malaria control strategies 

 These challenges have increased calls for innovative strategies particularly in areas with highest disease burden and to develop new and easy to implement control methods. 

 Community mass treatment with anthelmintic agents (i.e., dewormers) are community-based chemotherapy programs that have long been deployed as an effective tool for the control of major helminth infections in high burden communities. 

 The mortality of Anopheles mosquitoes, reduce sporogony, and also delay refeeding frequency. 

 Similarly, prolonged periods of mass treatment with ivermectin have been found to be linked to reduced mosquito feeding.

 Many studies have largely reported on the effects of ivermectin on Anopheles mosquitoes with a few reporting on the effects of other groups of anthelmintics or community treatment with any anthelmintics. In addition, many of the reports of the effects of other anthelmintics groups including the benzimidazoles and Praziquantel on malaria prevalence in observational settings have largely been conflicting. 

***Methods***

 This study was conducted using data obtained from a cross-sectional 

 There is a distinct rainy season that lasts from November – April, with a dry season from May – October and health seeking behaviors in Sussundenga, Mozambique from December 2019 – February 2020

 The village is divided administratively in 17 residential areas called “Bairros.” 

 Sussundenga village is the district capital where the central municipal offices and district level rural health center (RHC) reside. 

 A total of 2,837 households were identified in Sussundenga Village from the satellite imagery. A random sample of 125 households was taken with the goal of having 100 households for enrollment in the study and 25 households as backup for refusals and errors in the digitizing process. 

 Coordinates of the households were extracted using a GoogleEarth ProTM and maps of the selected households were created for the study team to conduct study visits.

 The study involved two visits to the selected households. The first was a notification visit where the study team introduced themselves to the head of the household and explained the objectives and procedures of the study. It is customary for the head of household to provide permission.

 Once the head of household gave permission, the study team conducted a household census with the head of household and began the process of individual informed consent with the household residents, for all adult (18+ years) residents and parental permission and assent from minors. 

 The only eligibility requirement was that the residents live in household full time. 

 The questionnaire also included questions regarding administration and timing of anthelmintic treatment. 

 The questionnaire was piloted among the study team to assess understanding and validity of the questions prior to the start of the study. A study nurse collected current malaria specific symptoms by self-report. 

 The results were recorded and, in the event, that a participant was positive for malaria, the study nurse referred them to the Sussundenga RHC for diagnosis confirmation and treatment. 

 Descriptive statistics were used to report continuous variables by medians and interquartile range. 

 A multivariable model was also used to investigate the association between any anthelmintic use and malaria infection. 

 use and malaria infection additional univariable and multivariable models were constructed with the main exposure defined as taking anthelmintic treatment in the previous 6 months. 

                                                                                                                                                     

***Results***

 Use of ITNs on the previous night was less prevalent among participants who previously.

  received anthelmintics (62%) compared to participants with no history of anthelmintics intake (71%) (Table 1). 

 The associations between various predictors and P. falciparum infection are presented in Table 2. Age, ITN use, and head of household’s full-time employment were independent predictors. 

 The odds of P. falciparum infection were 1.21 times higher for those who had ever taken anthelmintics compared to those who have never taken anthelmintics in the unadjusted model, however this lacked statistical significance. 

 In the unadjusted model, recent (< 6 months) intake of anthelmintics was associated with a 0.44 times lower odds of P. falciparum infection compared to those with non-recent intake of anthelmintics. 

 This result lacked in precision. 

***Discussion***

 Age of participants, use of ITNs, and head of household’s full-time employment were independent predictors of P. falciparum infection in Sussundenga village.

 However, the association lacked in precision and had wide confidence intervals. 

 Though our results lacked precision they showed that participants who received anthelmintics within the last 6 months had lower odds of P. falciparum infection compared to those that received anthelmintics over 6 months prior to the survey.

 Similar to our findings, Dila et al. in a meta-analysis found no significant association between treatment with anthelmintics and malaria prevalence at the end of follow up period (pooled OR 0.93, 95% CI: 0.62 - 1.38). Contrary to these findings, Sokhna et al. found an increase in malaria incidence among children presenting with concomitant helminths infection. Thereby, implying that an anthelmintic treatment was potentially associated with malaria risk in these settings.

 susceptibility to acute malaria by causing a major shift in the host immune response from Th1 to Th223 

 immune response to Th1 which ultimately lead to a decreased host’s susceptibility to acute malaria infection. 

 The timing of anthelmintics administration as well as the duration of helminth infection have emerged as critical determinants of a host’s immune response to P. falciparum parasites. 

 who received anthelmintics within a six-month period had lower odds of P. falciparum infection compared to those that received anthelmintics more than six-months prior, though this finding was not statistically significant  who received anthelmintics within six months had lower odds of P. falciparum infection than those who received anthelmintics more than six months prior. However, this finding was not statistically significant.

 Furthermore, the imprecision of our estimates was likely to have been from our small sample size as well as differences in mechanism of action and effects of the various anthelmintics that were received by participants on the overall immune response. Additionally, our study did not collect information on the different types of anthelmintics that were received by participants and the frequency of treatment. 

 Previous studies had found that repeated 2 – 4-monthly anthelmintic treatments can have a significant impact on Plasmodium infection.  Previous studies have found that repeated 2 – 4-monthly anthelmintic treatments can significantly impact Plasmodium infection.

 Our study has several limitations. First, this was a community- based cross-sectional survey and by design we were limited in our ability to establish any true causal relationship. Second, our small sample size of 277 may not have been large enough to detect true differences in this association as it was not powered for this analysis. 

 Third, we collected no information on specific classes of anthelmintics and dosages of anthelmintics received by the participants. This is important as pharmacological effects as well as effects on the level of immune responses could differ depending on the type of anthelmintics received. Lastly, malaria infection was measured by RDT only without confirmation with microscopy or polymerase chain reaction (PCR). This is a high transmission setting and some of the infections detected by RDT in this study were asymptomatic, but most were moderately symptomatic. However, it is likely that low density infections were missed using only the RDT. 

***Conclusion***

 These findings offer evidence for making decisions in planning mass community deworming and to inform the overall objectives of such policies.

Is the work clearly and accurately presented and does it cite the current literature?

Yes

If applicable, is the statistical analysis and its interpretation appropriate?

Yes

Are all the source data underlying the results available to ensure full reproducibility?

Yes

Is the study design appropriate and is the work technically sound?

Yes

Are the conclusions drawn adequately supported by the results?

Yes

Are sufficient details of methods and analysis provided to allow replication by others?

Partly

Reviewer Expertise:

Malaria epidemiology, malaria elimination, puyblic health

I confirm that I have read this submission and believe that I have an appropriate level of expertise to confirm that it is of an acceptable scientific standard, however I have significant reservations, as outlined above.

# Reviewer report: Reviewer response for version 1

**DOI:** 10.21956/wellcomeopenres.21654.r71938

**Published:** 2024-02-20

**Authors:** R Matthew Chico, Wilfred F Mbacham

**Affiliations:** 1 Associate Professor in the Department of Disease Control, London School of Hygiene & Tropical Medicine, London, UK; 2 President of Fobang Institutes / Chair of Graduate Programme, Unit for Life Sciences & Biol of Organisms, University of Yaoundé, Yaoundé, Cameroon

Akambase *et al.* have reported the results of a sub-group analysis conducted using data from a cross-sectional community-based survey designed to measure malaria risk, prevention, and health seeking behaviors in Sussundenga, Mozambique.  The authors had three objectives: (1) to identify the factors independently associated with *P. falciparum* malaria infection measured by rapid diagnostic test (RDT); (2) to determine whether prior intake of any anthelmintic agent was associated with risk of malaria infection; and (3) determine whether the timing of anthelmintic treatment impacted the association between treatment and malaria risk.  We applaud Akambase *et al. *for investigating co-infection and the potential interaction between infections and common interventions deployed to control them. There are, however, some important issues that need to be addressed (Main Comments) and other points (Minor Comments) that will help to improve the work.

**Main Comments:**

 1. The authors reported their results as if there had been evidence of effect when there was no evidence of effect. Consider contents from the abstract, specifically the Results section and the Conclusions. Underlining has been added for emphasis.

**Results**

*“There was no statistically significant association between prior receipt of anthelmintic and P. falciparum malaria infection after adjusting for age, ITN use and head of household full-time employment (OR = 1.37, 95% CI, 0.70–2.70, p = 0.36). However, recent intake of anthelmintics was associated with lower odds of testing positive for in the adjusted models (OR = 0.35, 95% CI, 0.07–1.80, p = 0.21), but this was not statistically significant.”*

**Conclusion**

*“Our findings show that the benefit of anthelmintics treatment as a control tool for P. falciparum malaria infection is likely tied to when it is administered rather than if it was ever administered. These findings offer evidence for making decisions in planning mass community deworming in sub-Saharan Africa.”*

 This discord between evidence and interpretation of clinical trial results has been described by (Boutron *et al.* [Ref 1]) as “spin” or a deliberate effort to misrepresent the findings.  We do not believe this has been the aim of Akambase *et al.* Regardless, more circumspect reporting is warranted.  The authors can refer to seminal work published by Boutron *et al. *on the topic of reporting non-significant study results, and by (Wang *et al [Ref 2]) *on sub-group analyses.  Pocock and Ware provide some helpful terminology for *p-*values.  Specifically, the authors should consult the figure entitled, “ *Six scenarios for primary endpoint of randomised trial comparing new and standard treatment groups*” for advisable phrasing related to the *p-*values they have.  A *p*-value of 0.21, as reported by Akambase *et al.*, reflects having no evidence of effect.  We acknowledge that Akambase *et al.* are presenting secondary endpoints from a cross-sectional community-based survey, and not a primary endpoint from a randomised clinical trial.  This difference in study design simply means that Akambase *et al.* should be even more cautious in their interpretation of results.

 2. Based on the points raised above, the title of the manuscript should not be, “Association of anthelmintic treatment with malaria prevalence in Rural Sussundenga, Mozambique.”  There is no association.  Thus, the title could be, “Assessment of anthelmintic treatment and malaria prevalence in Rural Sussundenga, Mozambique.”  Thereafter, throughout the manuscript, the authors should not refer to their findings as associations.  Similarly, the authors should not say their results “lacked precision” when there was no association.

 3. The Introduction section contains data and references that do not reflect the latest information available, e.g. case of malaria worldwide refer to 2020.  The authors should cite data from the 2023 World Malaria Report (2022 estimates 249 million cases).

 4. The authors state in the Introduction section that that, “global prevalence of malaria continues to increase,” and cites the 2021 World Malaria Report.  This statement is incorrect.  The global prevalence is not increasing.  However, the total number of cases is rising. This needs to be corrected.

 5. The authors describe how they arrived at their sample size, but they do not provide a power calculation and underlying assumptions for their sample size.  This is needed.

**Minor Comments:**

 1. There are some grammatical convention around the introduction and use of abbreviations that need to be applied:

 (a) Abbreviations should not be introduced unless they are going to be used again later in the main body of the text at least three more times. The authors should remove the following abbreviations, e.g. GTS, ACT, iPTP, IRB

 (b) Abbreviations should not be introduced in the abstract unless used again at least three more times in the abstract.  The authors should: (1) remove ‘RDT’; (2) write out insecticide treated net, odds ratio, confidence intervals.

 2. The term ‘easy to implement control methods’ should have hyphens, i.e. ‘easy-to-implement control methods’.

 3. *Plasmodium falciparum *should be written out in full the first time it is used and abbreviated as *P. falciparum *thereafter.  This should be done within the abstract and, again, in the main body of the text. 

 4. The term ‘data’ is plural and, therefore, phrasing should be ‘data are’ or ‘data were’ rather than ‘data is’ or ‘data was’.

 5. Table 1 should have a column of *p-*values to illustrate whether there were meaningful differences in characteristics between participants who had been de-wormed and those who had been de-wormed.

 6. We note that in an earlier paper by this research group, the authors were prompted by peer-reviewers to discuss the potential effect of *Plasmodium falciparum *histidine-rich protein 2 and 3 (PfHRP2/3) gene deletions on the accuracy of antigen-based rapid diagnostic tests for *Plasmodium falciparum* infection in their survey [4].  The authors responded by referring to a paper published in 2019 (having analyzed samples from 2018) and concluded, correctly in our view, that these gene deletions were not likely common in the survey area and, therefore, would not have affected their malaria-positivity rates as measured by antigen-based rapid diagnostic tests.  The authors should include some text related to this topic so that readers know the point has been given consideration.

Is the work clearly and accurately presented and does it cite the current literature?

Partly

If applicable, is the statistical analysis and its interpretation appropriate?

No

Are all the source data underlying the results available to ensure full reproducibility?

No

Is the study design appropriate and is the work technically sound?

Partly

Are the conclusions drawn adequately supported by the results?

No

Are sufficient details of methods and analysis provided to allow replication by others?

Partly

Reviewer Expertise:

Dr R Matthew Chico has over 25 years of global health experience in Latin America, Africa and the Asia-Pacific regions. His core expertise is in the design and conduct of impact evaluations and clinical trials aimed at improving maternal, reproductive, newborn and child health. His research focus is on expanding packages of care where doing so is supported by local epidemiology and operational capacity to address co-morbidities and reduce social inequities through the lifecycle.  Prof Wifred F Mbacham is a titular professor of Public Health Biotechnology.  His research focus is on the evaluation of implementation programs and the molecular basis of parasitism.  He also investigates host and pathogen genomics, and interaction between communicable and non-communicable diseases as comorbidities with inflammation as a unifying theme on these diseases.

We confirm that we have read this submission and believe that we have an appropriate level of expertise to state that we do not consider it to be of an acceptable scientific standard, for reasons outlined above.

## References

[1] : Reporting and interpretation of randomized controlled trials with statistically nonsignificant results for primary outcomes. *JAMA* .2010;303(20) : 10.1001/jama.2010.651 2058-64 10.1001/jama.2010.651 20501928

[2] : Statistics in medicine--reporting of subgroup analyses in clinical trials. *N Engl J Med* .2007;357(21) : 10.1056/NEJMsr077003 2189-94 10.1056/NEJMsr077003 18032770

[3] : Translating statistical findings into plain English. *Lancet* .2009;373(9679) : 10.1016/S0140-6736(09)60499-2 1926-8 10.1016/S0140-6736(09)60499-2 19375158

[4] : Modelling sociodemographic factors that affect malaria prevalence in Sussundenga, Mozambique: a cross-sectional study. *F1000Res* .2022;11: 10.12688/f1000research.75199.2 185 10.12688/f1000research.75199.2 35646333 PMC9131438
